# Grouting below Subterranean Water: Erosional Stability Test

**DOI:** 10.3390/ma14092333

**Published:** 2021-04-30

**Authors:** Jiří Boštík, Lumír Miča, Ivailo Terzijski, Mirnela Džaferagić, Augustin Leiter

**Affiliations:** Faculty of Civil Engineering, Brno University of Technology, Veveří 331/95, 60200 Brno, Czech Republic; mica.l@fce.vutbr.cz (L.M.); terzijski.i@fce.vutbr.cz (I.T.); karzicm@study.fce.vutbr.cz (M.D.); leiter.a@fce.vutbr.cz (A.L.)

**Keywords:** erosional stability, laboratory testing, grout mixtures, groundwater, test apparatus

## Abstract

The article is focused on the medium-term negative effect of groundwater on the underground grout elements. This is the physical–mechanical effect of groundwater, which is known as erosion. We conduct a laboratory verification of the erosional resistance of grout mixtures. A new test apparatus was designed and developed, since there is no standardized method for testing at present. An erosion stability test of grout mixtures and the technical solutions of the apparatus for the test’s implementation are described. This apparatus was subsequently used for the experimental evaluation of the erosional stability of silicate grout mixtures. Grout mixtures with activated and non-activated bentonite are tested. The stabilizing effect of cellulose relative to erosion stability has been also investigated. The specimens of grout mixtures are exposed to flowing water stress for a certain period of time. The erosional stabilities of the grout mixtures are assessed on the basis of weight loss (WL) as a percentage of initial specimen weight. The lower the grout mixture weight loss, the higher its erosional stability and vice versa.

## 1. Introduction

The objective of grouting a soil and rock mass is to create an “underground grout element” (UGE). This may have the function of reinforcement (support), sealing, or both. Possible complications arise from this basic objective of grouting works during grouting below the groundwater table. Simplifying somewhat, the results of grouting under the groundwater level are:

- UGE may not even be able to form;
- UGE may be able to form, but only with limited functionality;
- UGE may form with good functionality, but due to the subsequent action of groundwater, degrades gradually (to limited or zero functionality).

The reasons for the failed creation of an UGE (or insufficient functionality) can be different: for example, inappropriately selected type or character of the grout mixture, due to the porosity of soil, or inappropriately selected grouting procedure (e.g., low or high grouting pressure). These reasons and the contexts are usually discussed in the literature dealing with grouting, e.g., [1,2]. The implied reasons are usually also unambiguously unrelated to the presence or activity of groundwater.

Focusing only on the problems arising from grouting under the groundwater level, these problems logically result from the presence of underground water and its effect on the formation of or already-formed UGE. The negative effects of groundwater can be divided in terms of time to (this is a sub-division of the terms used by the authors of this article within their research work):

- short-term effects;
- medium-term effects; and
- long-term effects.

The short-term effects of groundwater (especially flow) on formed UGE are usually physical–mechanical; the grout mixture has not solidified enough. Most commonly, it is the segregation and/or flushing out grout mixtures from the UGE space. For example, this impact (authors label it as dispersion) is included in the research of Baluch et al. [3]. In this case, the dispersiveness of grout mixtures was tested by pouring grout mixture into a water-filled beaker. 

The medium-term effects of groundwater on UGE are usually physical–mechanical as well; however, compared to the short-term effects, there is no grout mixture dilution or flushing, but rather it is erosion. The formation of the UGE is mechanically disrupted and its function is negated as a result of the erosion process. The described state can occur by using grout mixture with a low erosional resistance (erosional stability) or by using a grout mixture in which the final erosional resistance is achieved very slowly (i.e., slow setting of the mixture). This process is in analogy with the internal erosion of soil by piping. This phenomenon has been investigated by many authors who studied it on soil [4] or soil treated by chemical stabilizers [5]. Research of silicate grout mixture erosion has not been found.

In the course of short-term and medium-term effects of groundwater, chemical degradation processes occur. In these cases, they are not of great significance as the grout mixture has been flushed out or was already eroded. 

The long-term activity of groundwater includes, once again, erosion. The possibility of suffusion cannot be excluded either. However, chemical degradation processes, which are commonly known as “corrosion”, may have the greatest importance. This is only applied when water contains corrosive substances. Wang et al. [6] described the damage of adding solids after seawater’s long-term corrosion, as well as analyses of its stress–strain curve and the relationship between damage variables and grouting solids.

The line between the time span of the above-mentioned categories is not fixed, which obviously arises from the above-mentioned facts. It depends, for example, on the speed of the solidifying and hardening processes of the grout mixture, on the hydraulic gradient or on the types and amounts of chemical substances present in the water. An order-of-magnitude estimate of the time span is:

- short-term effect—from tens of minutes to a low number of hours after the application of the grout mixture;
- medium-term effect—from a low number of hours to tens of hours after the application of the grout mixture;
- long-term effect—more than hundreds or thousands of hours after the application of the grout mixture.

For example, in the experiments described below, the line between the short-term and medium-term effects can be estimated to be between 5 and 6 hours after the production of the grout mixture. Within 5 h of its production, the grout mixture was flushed out as a result of the effect of flowing water. 

The danger arising from physical–mechanical processes is the largest for grouting of highly permeable and water-bearing horizons. This is where a significant negative underground grout element process of disruption by ground water may be expected. For low permeability horizons (though water-bearing), the intensity of groundwater flow is, from the given perspective, naturally insignificant.

Erosion is caused by the mechanical effects of the surrounding substance’s movement (in this case flowing water); therefore, the erosional resistance of grout mixtures can be deduced from their strengths. However, there is no general relation between strength and erosional resistance. This is understandable, since there is no "standard" intensity of erosive action, nor are there "standard" conditions. In addition to measuring the strength, there are non-standard tests of erosional resistance that attempt to directly simulate the process of erosion. However, these are single-purpose devices to a limited extent.

For laboratory testing of grout mixture resistances, it is possible to use both mentioned principles. The first is to monitor the strength of grout mixtures. Applied shear strength measurements (e.g., the penetration method) are the best for the monitoring of relatively low-strength "young" grout mixtures. The second method for determining the erosional resistance is a test of erosional stability. This test measures the resistance of the grout mixture (grouted element) to mechanical deterioration caused by flowing water.

The description of the erosional stability test, technical solutions and implementation of test apparatus is the main subject of this article. This test was subsequently used for the experimental evaluation of the erosional stability of silicate grout mixtures.

## 2. Overview of Laboratory Test Apparatus for Erosional Stability Testing of Geomaterials

Studies on internal erosion originally focused on a mechanical principle, where particle and opening sizes in the soil that allow particle movement were primarily investigated [7,8].

Over the years, a number of different laboratory methods have been developed in order to test soils according to their susceptibility to erosion, e.g., the pinhole test, flume tests, the jet erosion test and the rotation cylinder test. These tests have been summarized by various authors, e.g., Wan and Fell [9,10].

The rotating cylinder test (RCT) was developed by Moore and Masch [11]. The RCT uses a block of soil, suspended and submerged, inside a rotating cylindrical chamber, where the rotation of the cylinder induces a flow around the specimen which causes erosion. Torque is applied to the specimen and the erosion rates are measured. The results are used to estimate applied stress and erodibility parameters.

Arulanandan et al. [12] extended previous research by performing erosion tests on samples by circulating collected water on undisturbed samples inserted into a hydraulic flume from the bottom. The flume test is used for modeling erosion mechanism, where water is flowing parallel to the soil surface at a certain speed and depth. The erosion rate is only visually observed and described in most cases, while hydraulic shear stress on the soil surface is deduced from the flow velocity and water depth.

Lefebvre et al. [13] and Rohan et al. [14] developed the drill hole test (DHT). The DHT is conducted by applying a flow rate-controlled pressure flow to a cylindrical clay sample using a predrilled axial hole. The friction head loss along the specimen is measured to determine shear stress and the collected eroded material is used to calculate the erosion rate.

A special type of flow-over-surface test is the erosion function apparatus (EFA) developed by Briaud et al. [15]. The EFA test uses site-specific soil samples acquired via thin-walled tubes to generate an erosion rate and shear stress. During the EFA test, a data acquisition system records the velocity and amount of soil eroded. These data are used to calculate the erosion rate and shear stress.

The jet erosion test (JET) was developed at the Agricultural Research Service Hydraulic Engineering Research Unit, Stillwater, Oklahoma [16]. This method is applicable to a wide range of soils. JET can be performed in situ [17,18] or in a laboratory [19] using tube samples or remolded samples in compaction molds. Testing has been successfully carried out on specimens as small as 75 mm (3 inches) in diameter and uses a submerged hydraulic jet to produce scour erosion. JET estimates the critical shear stress needed to initiate erosion.

The hole erosion test (HET) was developed by Wan and Fell [10] to measure the erosional properties of soils. HET involves measuring an accelerating flow rate through an eroding pre-formed hole in a test specimen. HET enables the estimation of the critical shear stress and erosion rate coefficient. All test results of the hole erosion tests give an interesting relationship between the critical stress and the erosion rate coefficient. A greater critical stress implies a greater erosion rate index (i.e., slower erosion). 

Sanchez et al. [20] were the first to develop an internal erosion test within a modified triaxial apparatus to evaluate the erosion of core embankment materials. Recent experiments by Bendahmane et al. [21] revealed the complex effects of confining pressure on internal erosion. The triaxial erosion test was conducted by Bendahmane et al. [21,22] to measure the effects of internal flows on sand/kaolin samples.

Richards and Reddy [23] developed a true triaxial system to investigate the piping potential of both cohesive and non-cohesive soils. A computer-controlled triaxial testing apparatus was modified to allow the independent control of hydraulic gradient and stress state for investigating the initiation and development of soil internal erosion, and the stress–strain behavior of soil subjected to internal erosion.

One of the most recently presented pieces of experimental apparatus is the cross erosion test (CET) [24,25], which is devoted to the measurement of the initiation of suffusion. The test consists of a clear water injection into a first drill hole, and the recovery of washed-out particles suspended in water in another drill hole. This technique can be portable, in order to consider the in situ risks of internal erosion at dams and dikes.

All the mentioned methods, in most cases, include a circular cross-sectional shape of the specimen. The position of the specimen during the test with regard to the direction of water flow is mainly vertical, except for in the HET test, where it is horizontal. Monitoring of piezometric height takes place using a piezometer or manometer. 

The evaluation of erosional resistance is mainly based on quantifying the amount of eroded material; specifically, the amount of washed-out material in the outflow and hydraulic gradient. Internal erosion in an internally unstable soil will occur when the hydraulic gradient exceeds a certain critical value. 

As stated earlier, a summary of the findings from the literature indicates that quite considerable attention has been devoted to experimental soil erosion modeling. However, experimental modeling of erosion, especially of grout mixtures, is not often mentioned in the literature. One method of laboratory testing of erosional resistance is the principle described by Verfel [1]. The test principle is basically the same as that for determining soil erosion, i.e., the grout mixture (partially stiffened) is exposed to water flow stress. Here, the erosional resistance is tested on a specimen of grout mixture with a height of 70 mm, which is cast into a cylindrical vessel (the test cell). A hole is formed in the specimen by inserting a glass stick with a diameter of 8 mm during preparation of the specimen. The glass stick is removed after 18 h and water is fed into the upper cell space (above the upper specimen surface) under such a pressure as to form a gradient that causes water flow through the coaxial hole in the specimen at a velocity of about 2 ms^−1^. The result of this laboratory test is weight loss of the grout mixture, which is eroded by the water flowing through a hole in the specimen for one hour.

The erosion tests are also mentioned in Austrian standard ÖNORM B 4452 [26]. The laboratory testing process of the erosion test and schematic drawings and the dimensions of the test cell (specimen) are described in the appendix of this standard. The principle of the test is also based on investigation of hole erosion behaviors. A cylindrical hole is formed in the axis of the cylindrical test specimen from the cut-off wall material. The hole’s diameter in this case varies from 1–10 mm. The specimen is placed into a test cell, which is connected to a water-pressure system during the test.

## 3. Experimental Verification of Erosional Stability of Grout Mixtures

The development of the research framework carried out at the Faculty of Civil Engineering, Brno University of Technology, included the method for testing the erosional stability, according to Verfel [1]. We decided to design and develop our own apparatus, since there is no standardized method for testing at present, and it was not possible to purchase an appropriate laboratory apparatus.

### 3.1. Apparatus and Test Process

**Figure 1:**
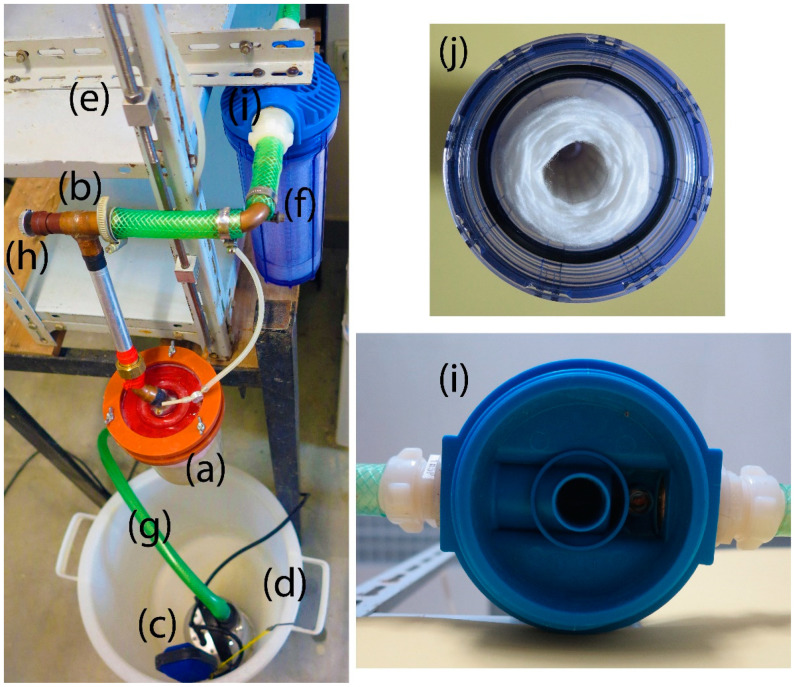
Test apparatus for determining erosional stability: (**a**) test cell; (**b**) flow controller; (**c**) pump; (**d**) storage/recycling vessel; (**e**) supporting structure; (**f**) filter; (**g**) supply pipe; (**h**) control button; (**i**) cap of the filter; (**j**) opened filter view.

The test apparatus for determining erosion stability consists of several basic structural units (Figure 1):

- storage (recycling) vessel;
- flow controller;
- test cell;
- filter;
- pump; and
- supporting structure.

The storage vessel accumulates a test fluid (water) and enables its recycling. There is a submersible pump placed in the vessel that is connected to the flow controller via a pipe. The flow controller is used to maintain constant hydraulic conditions (i.e., constant volume flow rate of 0.1 L/s at a water flow velocity of 2 ms^−1^) through the test cell. If needed, it is possible to increase or decrease the volume flow rate using the control button, which is on the flow controller. The solid particle filter is part of the test circuit. The filter captures particles of the tested matter that erode during the test, and any other impurities that may occur in the circuit.

The test cell contains a specimen of the grout mixture. The cell has two parts. The grout mixture specimen is placed in the lower part of the cell. There is an 8 mm round hole in the bottom. The upper part is a pressure cap and both parts can be sealed together. The cap is equipped with an inlet fitting, flow straightener and air vent. The diameter of the test cell in the bottom is about 100 mm; the cell slightly widens upwards conically. A test cell holder enables the cell position to be adjusted.

**Figure 2:**
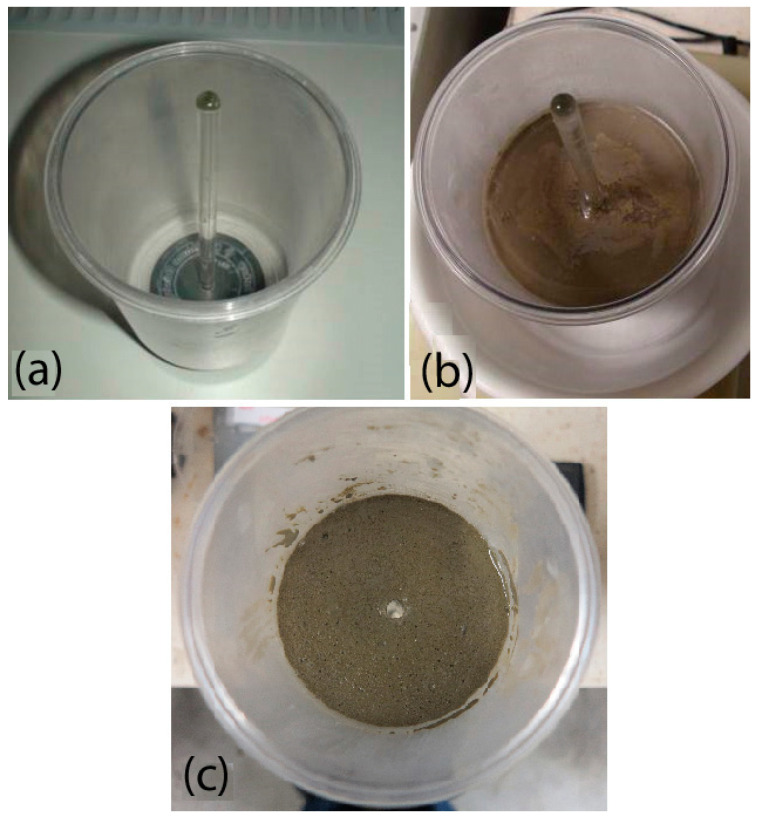
Specimen preparation: (**a**) empty test cell; (**b**) test cell with grout mixture; (**c**) specimen ready for testing.

The test specimen is prepared by casting grout mixture into the test cell. A glass (or plastic) stick with a diameter of 8 mm is inserted into the hole at the bottom of the test cell (prior to casting). The specimen height in the cell should be about 70 mm at the time of starting the test (Figure 2). The glass stick is removed before starting the test, and there is an 8 mm round hole that remains in the bottom of the specimen. The hole in the bottom of the test cell is temporarily sealed (glued) in the next step, and the upper space of the grout mixture is flooded with water. After the cell is closed, it is placed in the cell holder and connected to the flow controller via a pipe. The flow controller must be adjusted so that the water flow velocity through the non-eroded hole in the axis of the specimen is about 2 ms^−1^.

According to the standard procedure described in [1], tests should be initiated after 18 h of grout mixture production. However, within the demonstration examples shown in Section 3.2 and Section 4, during the erosional stability test in compliance with the prescribed time delay (18 h) from the grout mixture preparation to the beginning of the test, there was no specimen disruption caused by flowing water. Since this is a purely comparative test of grout mixture, this step was necessary to change the conditions of the test to detect potential differences between suspensions (i.e., to capture the potential improvement expected for modified grout mixtures). It turned out that, technically, the easiest way was to change the time delay. This was based on the fact that the age of a specimen must allow for the creation of the desired hole in the specimen, and the measurement of the weight loss after the test. After several experiments, the time delay was set to six hours. This kind of test can only be taken as characteristic for properties of UGE if the grout mixture is appropriately stable. A stability limit of 2% for increasing the grout mixture’s specific weight between its casting and the beginning of the water flow was used (as recommended).

**Figure 3:**
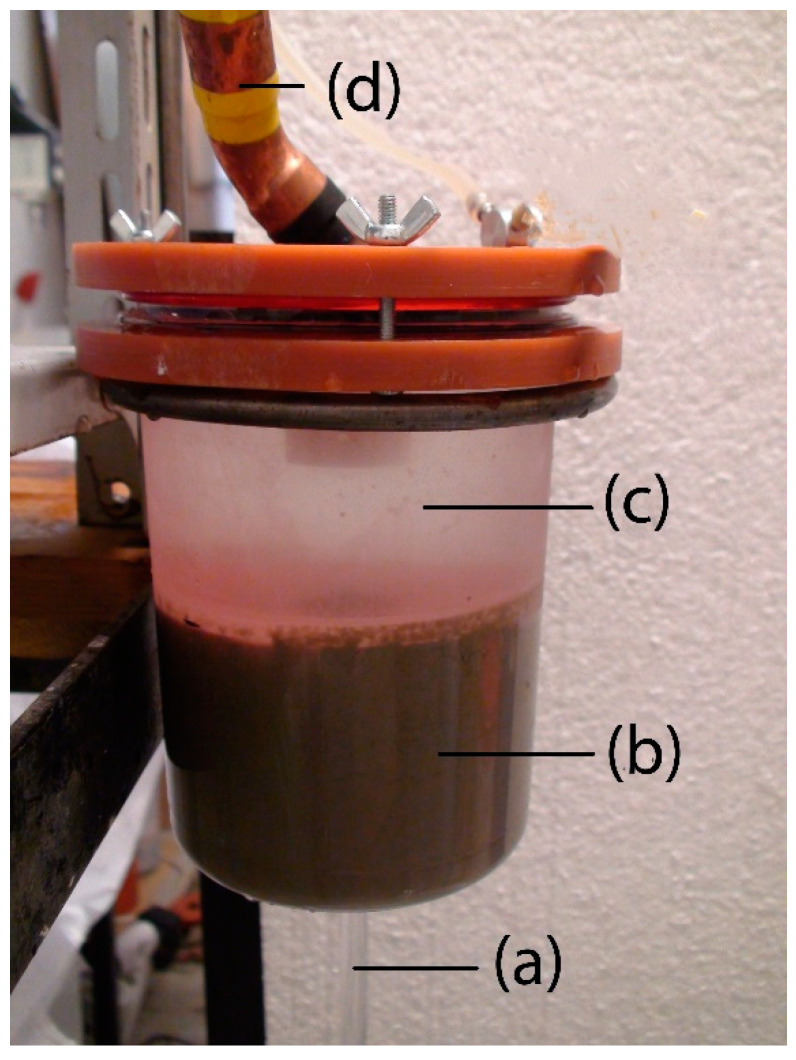
Grout mixture specimen during testing: (**a**) effluent water; (**b**) specimen of grout mixture; (**c**) test cell; (**d**) supply pipe.

The water flows into the test cell (Figure 3) through the filter and the flow controller from the storage/recycling vessel during the test. The velocity of water flowing during the tests was kept constant at the value of 2 ms^−1^. Drinkable water from the Brno aqueduct was used for these experiments.

The test cell is equipped with a flow straightener. This is a specially shaped inlet that forces the inflowing water jet through a large number of small holes. Such a small diameter of the outflowing water jet formed flooded stream and also ensured good venting. Air bubbles can be purged through the air vent in the cap of the test cell. Undisturbed water then flowed into the hole in the specimen. The water (with possible eroded particles of the specimen) falls into the storage/recycling vessel. The height of the test cell above the storage/recycling vessel was set to allow flow rate monitoring using the volumetric method. If the erosional effect is negligible and therefore the water is clear, it can be reused. Water reuse is permitted by a submersible pump in the storage/recycling vessel. This eliminates the use of tap water. The test was terminated after one hour of water flowing through the specimen.

The erosional stability test was evaluated by recording/verifying the following: specimen weight before and after the test, the water flow rate, and the elapsed time. The erosional stability of the grout mixture was assessed on the basis of weight loss (WL) as a percentage of the initial specimen weight. The lower the grout mixture weight loss (WL), the higher its erosional stability (erosion resistance) and vice versa. A WL value equal to 0, or very close to 0 (cases in which only the inlet edge of the hole in the specimen is impacted), means that failure of the grout mixture in the allotted time did not happen.

### 3.2. Application of the Erosional Stability Test—Examples of Experiments

The test described in the previous section and the created laboratory apparatus were used for subsequent tests. These activities were primarily focused on finding ways to reduce the negative physical–mechanical effects of flowing groundwater on the underground grout elements created by grouting. Here, further focus was only on the testing of selected options, leading to an increased erosional stability of grout mixtures.

Grout mixtures based on silicate composites were the only subjects tested. From a broader set of these grout mixtures, clay–cement sealing mixtures were selected. The grout mixtures were prepared on the basis of Na^+^ ion-activated bentonite, commercially designated as Bentovet K (Gemerská nerudná spoločnosť, Hnúšťa, Slovakia) and also on non-activated bentonite, commercially designated as Bentonit 75 (manufacturer: Keramost, a.s., Most, Czech Republic).

**Figure 4:**
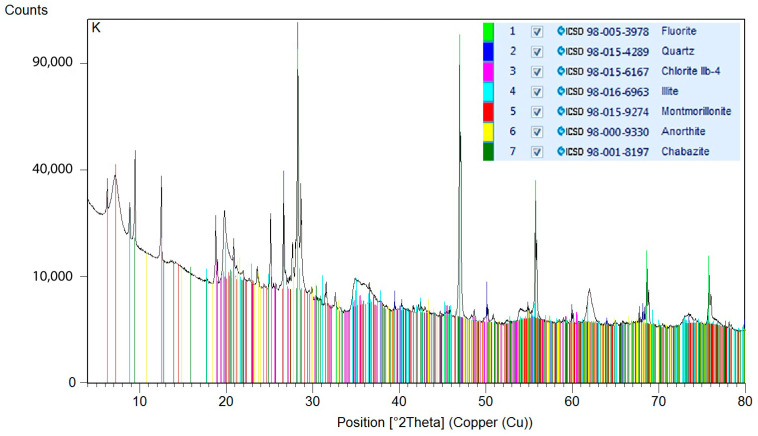
Diffractogram of Bentovet K.

**Figure 5:**
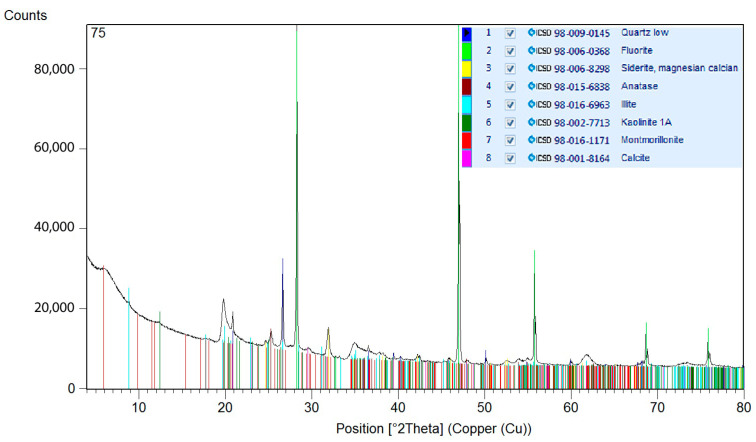
Diffractogram of Bentonit 75.

For determination of the mineralogical compositions, bentonite samples were subjected to X-ray diffraction analyses. The specimens were crushed prior to analysis in isopropanol using the McCrone Micronising Mill with the addition of 20 wt.% of an internal standard (i.e., after addition: 80% of specimen + 20% of standard). The used standard was fluorite (CaF_2_). The addition of the internal standard allowed the quantification of the amorphous phase. X-ray diffraction (XRD) analyses were performed on a Rigaku Smartlab apparatus with a Cu-anode (λKα = 0.15418 nm), 2D position-sensitive detector used in the 1D mode and fixed divergence screens in conventional Bragg-Brentano parafocusing Θ-Θ reflection geometry. The measured angular area was 5–80 °2θ. The step size was 0.013° 2θ and the scan step time was 255 s. Total measurement time was 5769 s. The data were processed using Panalytical HihgScore 3 plus software. Quantitative phase analyses were performed using the Rietveld method. The structural patterns from the database of ICSD 2012 were used to refine the structural specification and for quantification. The results of the quantitative phase analyses are listed in Table 1. The overall compositions of the specimens, including the content of the amorphous phase, were quantified, as was the composition of the isolated crystalline part. Diffractograms are shown in Figure 4 and Figure 5.

Portland composite cement CEM II/BM (S-LL) 32.5 R (manufacturer: Českomoravský cement, a.s., Mokrá, Czech Republic) was used for the laboratory preparation of the grout mixtures.

The laboratory production of the grout mixture followed common preparation methods used in practice. A two-stage process, as well as a single-stage process, of grout mixture preparation was used. The two-stage process of preparation was only used for grout mixtures based on Bentovet K. The single-stage process of preparation was used for the grout mixtures based on Bentovet K and for the grout mixtures based on Bentonit 75. The two-stage process for grout mixture preparation was as follows:

- The water bentonite mixture (BM) was prepared, mixing time was 10 min.
- The BM was left to cure in a closed vessel for approximately 24 h at a temperature of 20 ± 2 °C.

- The grout mixture itself was prepared by adding cement to the cured BM while stirring continuously. The stirring time was 5 min in this case.

The single-stage process of the grout mixture preparation was as follows:

- The water bentonite mixture was prepared—it was mixed for 10 min (grout mixtures on the basis of Bentovet K) or for 5 min (grout mixtures on the basis of Bentonit 75).
- Cement was added gradually to the BM while mixing continuously. It was mixed for 5 min in this case.

Mixing of the grout (both two-stage process and single-stage process) was accomplished through the use of a laboratory stirrer (Heidolph RZR 2020). A dissolver stirrer with a diameter of 100 mm and revolutions of 1300 min^−1^ was used. 

After cellulose was added to the finished mixture, stirrer revolutions were reduced to 400 min^−1^ and the process of stirring took place for a further 1 min.

In the first stage of the experimental work, the so-called “standard grout mixture” was designed and tested. The composition of the grout mixture (1 m^3^) was as follows: 42.6 kg of activated bentonite (dry weight), 350 kg of cement, and water for the remainder. This was a grout mixture recipe that was created at the Brno University of Technology previously, having been actually utilized for sealing works by the BauGeo company. In the next phase of the experimental work, design and testing of the modified grout mixtures were carried out. The compositions of the modified grout mixtures were modified with the aim of increasing erosional stability.

Some designed and subsequently tested modifications included, e.g., an increase in bentonite content in the grout mixture, using a different type of bentonite in the grout mixture, or the use of special additives to enhance grout mixture thixotrophy.

## 4. Results and Discussion

### 4.1. Increase in Bentonite Content in the Grout Mixture (Example One)

The standard dose of Bentovet K was 42.6 kg (dry) per 1 m^3^ of initial bentonite mixture (i.e., the standard grout mixture with Bentovet K labeled as K1). In an effort to improve the grout mixture’s erosional stability, the Bentovet K dose was gradually increased to 50, 57.5 and 65 kg per 1 m^3^ of bentonite mixture. The dosages used during the two-stage process of grout mixture preparation are shown in Table 2.

The used Bentovet K concentration range covered the entire useful range of this grout mixture type. If the minimum concentration was used, an unstable grout mixture is expected. If greater than the maximum concentration was used, the grout mixture is difficult to pump and inject. The cement dosage in all tested mixtures was constant: 350 kg/m^3^ of grout mixture. The impact of these changes on the grouts’ erosional stability was observed.

**Figure 6:**
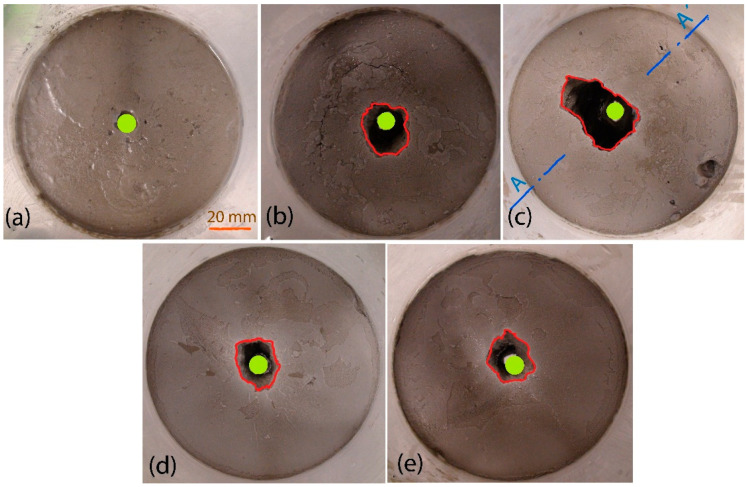
Inlet side of specimens with Bentovet K: (**a**) standard grout mixture K1 before the test; (**b**) grout mixture K1 after the test—WL = 4.7%; (**c**) grout mixture K2 after the test—WL = 11.4%; (**d**) grout mixture K3 after the test—WL = 2.1%; (**e**) grout mixture K4 after the test—WL = 5.0% (the edge of the failure area in red, the initial hole in green).

**Figure 7:**
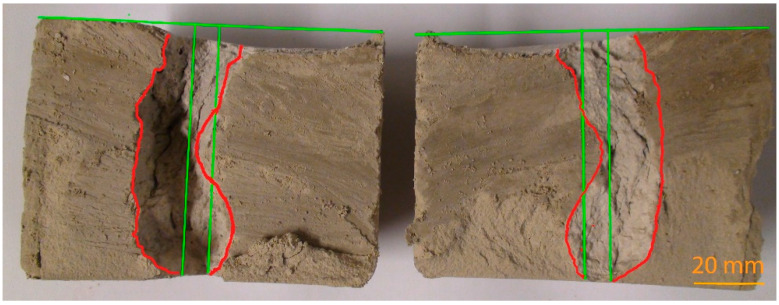
Specimen cross section A–A’ after the test (see Figure 6c)—grout mixture K2 based on Bentovet K (the edge of the failure area in red, the initial hole and inlet side in green).

An example of erosional damage can be seen in Figure 6. This represents the inlet side of the specimens before (a) and after the tests (b, c, d, e), and the confirmed percent value of weight loss of the grout mixture (WL). The contour of the hole in the specimen before (green highlighted) and after (red highlighted) the tests is marked in Figure 6. In Figure 7, the specimen’s cross section after the test of K2 grout mixture with the lowest erosional stability is presented. Again, the location of the hole contour in the specimen and the inlet side before the test is highlighted in green. The edge of the failure area after the test is highlighted in red. It is obvious from the figure that the decreasing erosional stability of the grout mixtures causes significant distortion of the specimen’s inlet edges. This phenomenon is accompanied by a widening of the hole in the specimen. It is clear that the extent of the widening depends on the value of WL. The higher the WL value, the wider the hole, and vice versa. Concurrently, the hole is widened along the full height of the specimen and it can be noticeably asymmetric (with regard to the longitudinal axis of the specimen), see Figure 7.

The erosional stabilities of these grouts appear to be uncorrelated with the used bentonite dose (within the given range). This is not surprising, since in the case of erosional stability, the most significant factor is the strength of solidified grout, which is in the test time of 6 h, determined by the amount and type of cement, respectively, using the water/cement ratio. These parameters were constant in a given series of grout mixtures (cement dose), or changed insignificantly (water/cement ratio), see Table 2.

### 4.2. Use of Different Bentonite Type in the Grout Mixture (Example Two)

Chemically activated (natrificated) bentonites are commonly used in grout mixtures (Bentovet K was used in this study—see example one). This means that the natural Ca form of the main bentonite mineral, montmorillonite, is artificially converted to the Na form during the natrification process. This form has significantly improved solvating ability. If the suspension of natrificated bentonite is mixed with cement, the hydration of cement releases Ca-ions that, in turn, convert the suspended Na-bentonite (montmorillonite) back to the Ca form. The stabilizing ability of bentonite, therefore, decreases over time. However, simultaneously, the gel phase and later the crystalline phase of Al, Ca and Fe hydrosilicates from the cement are formed. Therefore, the mixture gradually solidifies and hardens. An ordinary well-designed grout mixture maintains its fluidity for a relatively long time period before strengthening processes gradually take over.

The aforementioned processing scheme is not suitable for most mixtures with increased erosional stability. If we replace the natrified bentonite in the grout mixture with Ca-bentonite, the dose must be, given its lower solvating capacity, significantly higher (see Table 2 and Table 3). Generally, depending on the parameters of both forms of bentonite, the dose may increase by a factor of three to five. The advantage for such a suspension is that the Na and Ca ions are not reversely interchanged, e.g., the strengthening processes start significantly faster. The disadvantage is the higher price of the total doses of Ca-bentonite in grout mixtures.

The two variants of grout mixtures based on chemically non-activated (i.e., in the Ca-form) bentonite (Bentonit 75) were prepared and tested. The used bentonite concentration range was again chosen to cover the entire useful range of this type of grout mixture (from instability to difficult pumping and injection). Due to the bentonite characteristics, considerably higher doses of bentonite were used. The particular dosages used during a single-stage preparation process are shown in Table 3.

**Figure 8:**
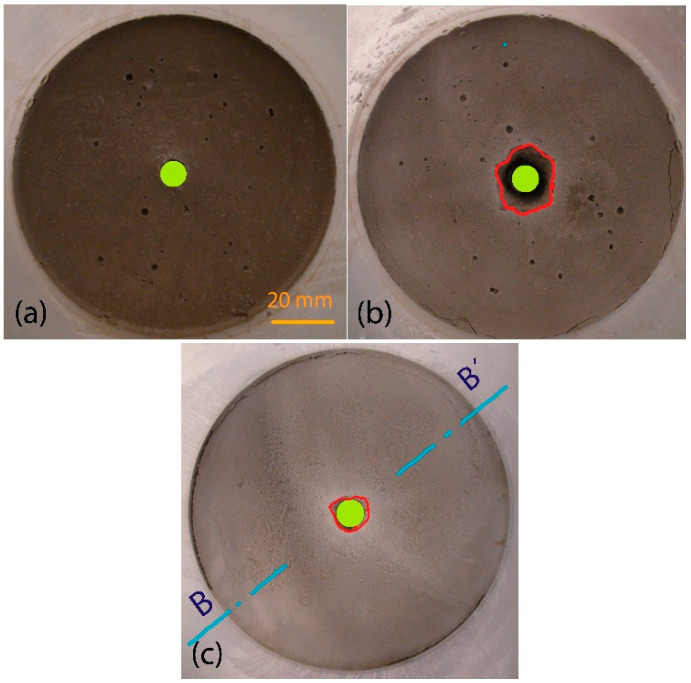
Inlet side of specimens with Bentonit 75: (**a**) grout mixture B1 before the test; (**b**) grout mixture B1 after the test—WL = 1.4%; (**c**) grout mixture B5 after the test—WL = 0.2% (the edge of the failure area in red, the initial hole in green).

The erosional stability in the case of a grout mixture based on non-activated bentonite (see Table 3 and Figure 8) appears to be significantly higher (i.e., the WL is lower) than that of a grout mixture based on activated bentonite. Using non-activated bentonite or increasing its concentration in the grout mixture is a simple solution that can be easily implemented in practical applications.

**Figure 9:**
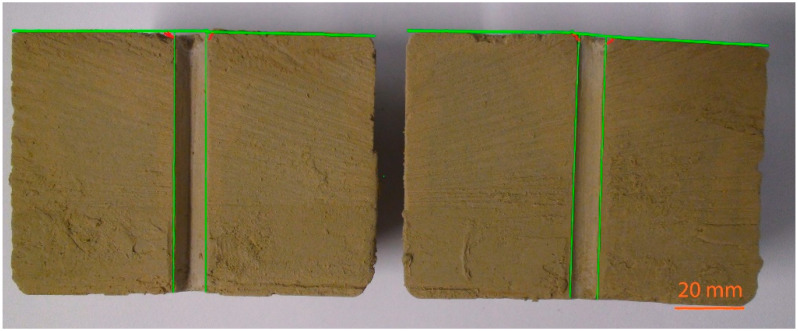
Specimen cross section B–B’ after the test (see Figure 8c)—grout mixture B5 based on Bentonit 75 (the edge of the failure area in red, the initial hole and inlet side in green).

The inlet side of the specimens before (a) and after the test (b and c) are shown in Figure 8. The vertical cross section of the grout mixture B5 specimen after the test is shown in Figure 9. The contour of the hole before and after the test is marked as in the case of the grout mixture based on Bentovet K. It is apparent that erosion only occurs at the inlet edge of the hole of the specimen. However, in the case of grout mixtures based on activated bentonite (see, e.g., Figure 7), there is a significant distortion of the hole along the entire specimen length.

This can be explained by the fact that the suspension has significantly higher strength after six hours. This is due to the significantly lower water/cement ratio compared to the grout mixtures with activated bentonite (e.g., 2.35 for grout mixture B1 or 2.48 for grout mixtures K3; see also composition of grout mixtures in Table 2 and Table 3).

### 4.3. Use of Additives to Enhance Grout Mixtures’ Thixotropy (Example Three)

To increase the yield stress and thixotropy of the grouts, cellulose derivatives can be used. In addition to the formerly obligatory carboxy-methyl-cellulose (CMC), a number of modified derivatives are available today that have improved dispersibility and solubility in water, with varying efficiencies. We used Culminal C9133 cellulose, which is a methyl-hydroxy-propylene cellulose with a viscosity of the standard (2%) solution in water of 4500–6500 mPa.s. It is suitable for application in systems with a large number of dispersed substances.

Several variants of grout mixtures based on Bentovet K and Bentonit 75 were prepared with different concentrations of cellulose in the grout mixture (0.5% and 0.75% of the cement weight). The cement dosage in all tested grout mixtures was constant; again, 350 kg/m^3^ of the grout mixture.

**Figure 10:**
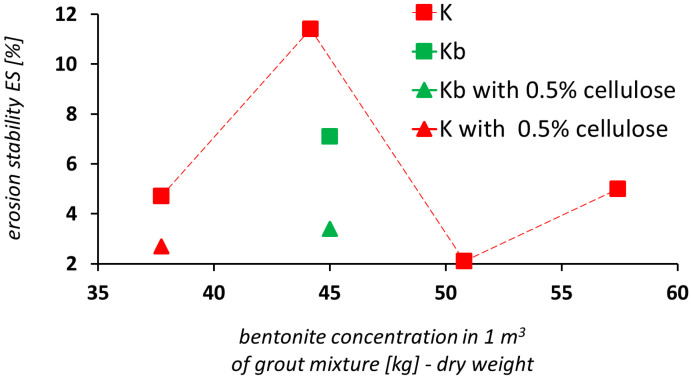
Weight loss of grout mixture (WL) vs. bentonite concentration: grout mixtures based on Bentovet K (K—two-stage process of grout mixture preparation, Kb—single-stage process of grout mixture preparation).

**Figure 11:**
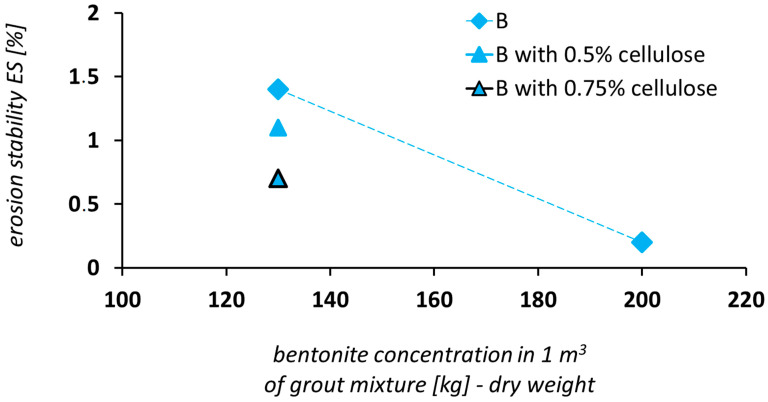
Grout mixture based on Bentonit 75: weight loss of grout mixture (WL) vs. bentonite concentration.

When cellulose is added to the grout mixture, there is an expected increase in the yield stress and thixotropy. It has a positive effect on the erosional stability of the grout mixture as it increases substantially (Figure 10 and Figure 11). Concerning grout mixture on the basis of activated bentonite, there was a verified increase by a factor of 1.7 in the erosional stability after 0.5% of cellulose was added (a decrease in the WL value from 4.7% to 2.7% for the K1 grout mixture) and an increase by a factor of 2.1 in the erosional stability (a decrease in the WL value from 7.1% to 3.4%) for the K2 grout mixture (single-stage process of the grout mixture preparation, designated as Kb in Figure 10).

Regarding grout mixtures on the basis of non-activated bentonite, there is no such increase in erosional stability as that in grout mixtures based on activated bentonite. There was an increase by a factor of 1.3 in the erosional stability of the grout mixture after 0.5% cellulose was added to the B1 grout mixture. It corresponded to a decrease in the WL value from 1.4% to 1.1%.

There was an increase by a factor of 2.0 in the erosional stability of the grout mixture after 0.75% cellulose was added to the B1 grout mixture. This corresponded to the decrease in the WL value from 1.4% to 0.7%.

The stabilizing effect of these molecular chains is temporary; therefore, these measures should be combined with those ensuring rapid solidification of grout mixtures (e.g., the use of non-activated instead of activated bentonite).

## 5. Conclusions

The erosional stability test is one way to allow for the evaluation of medium-term resistance of a grout mixture to the mechanical influence of flowing water. The authors are not aware of any published papers and results on erosion tests of silicate grout mixtures. From this point of view, we can consider these experimental works as the first of their kind.

A new laboratory apparatus for performing the test was presented here. The apparatus was designed for the purpose of comparatively evaluating grouts’ erosional resistances:

- The principal and subsequent evaluation of the erosional resistance was based on the study of Verfel (1992);
- Erosional stability was quantified by the relative weight loss of a grout (WL) during the test. Grout mixture, which is resistant to erosion, has a WL value equal to 0. This is a condition for real practical use;
- The apparatus is useful for practical use in ordinary laboratory conditions due to its operational and structural simplicity;
- This also allows a visual assessment of the extent of the erosional impact on a specimen. For example, only the inlet edge of the hole in the specimen is impacted, etc.;
- The enhanced apparatus design allows, if necessary, for erosion stability determination for different boundary conditions than those specified by Verfel (1992), or those that selected during the performed tests. In particular, there is the possibility of water flow control and an option to choose the grout mixture specimen height in the test cell. Alternatively, a simple irreversible modification (enlargement) of the hole in the test cell (grout specimen) is possible.

In addition, the use of this apparatus was demonstrated in some tests. The erosional stabilities of the grout mixtures were evaluated 6 h after production. It was confirmed that the choice of bentonite type, adjusting a bigger dose of non-activated bentonite, or the use of cellulose in the grout mixture may lead to a change in the grout mixture erosional stability. Based on the results presented in the paper, it can be stated that:

- A substantial increase in the erosional stability of grout mixture was reached by replacing activated bentonite with non-activated bentonite. It appeared that there was a decrease in WL value even to 0.2, i.e., the value was very close to 0;
- By increasing the dose of non-activated bentonite in the grout mixture, the erosional resistance of the grout mixture was increased. A 54% increase in the bentonite dose led to decrease of WL of seven times. Using non-activated bentonite (alternatively an increase of its concentration in the grout mixture) is a measure, which does not lead to a more complex composition of the grout mixture (or it does not require more complex preparation process of the grout mixture). Such a solution can be easily implemented in practical applications;
- Concerning the grout mixture on the basis of activated bentonite, the erosional stability seems not to correlate with the bentonite dose used (within the tested range). The value gains were in the range between 2.1 and 11.4;
- The addition of cellulose to the prepared grout mixture results in a positive effect on the erosional stability, i.e., there was a decrease in WL. The 0.5% cellulose concentration in the grout mixture led to a decrease of WL of 2.1 times. However, the stabilizing effect of cellulose was temporary; therefore, these measures should be combined with those ensuring rapid solidification of grout mixtures.

## Figures and Tables

**Table 1:** Results of quantitative phase analysis.

Specimen	Bentonit 75		Bentovet K	
Mineral	Specimen Composition Including Amorphous Phase [%]	Specimen Composition of Crystalline Part Only [%]	Specimen Composition Including Amorphous Phase [%]	Specimen Composition of Crystalline Part Only [%]
Montmorillonite	50.8	63.5	51.1	67.1
Quartz	8.3	10.4	7.6	10.0
Illite	5.1	6.3	8.3	10.9
Kaolinite	1.2	1.5	-	-
Calcite	0.2	0.2	-	-
Siderite	10.6	13.2	-	-
Anatase	4.0	4.9	-	-
Feldspar (plagioclase)	-	-	1.3	1.7
Chabazite	-	-	1.3	1.7
Chlorite	-	-	6.5	8.6
Amorphous phase	19.8	-	23.9	-

**Table 2:** Composition and WL of grout mixtures based on Bentovet K.

Indication	Bentonite Mixture (BM) *		Grout Mixture *		Bentonite Concentration [% of the Grout Mixture Weight]	Weight Loss (WL)
	Bentovet K (Dry Weight) [kg]	Water [kg]	BM [kg]	Cement CEM II/B-M (S-L)/32,5R [kg]		[%]
K1	42.6	984.2	907.8	350.0	3.0	4.7
K2	50.0	981.5	910.7		3.5	11.4 **^+^**
K3	57.5	978.7	915.1		4.0	2.1
K4	65.0	975.9	919.6		4.5	5.0

* Bentonite (grout) mixture composition is specified per m^3^. ^+^ Arithmetical average from two tests (12.5% and 10.2%).

**Table 3:** Composition * and WL of grout mixtures based on Bentonit 75.

Indication	Bentonit 75 (Dry Weight) [kg]	Cement CEM II/B-M (S-L)/32,5R [kg]	Water [kg]	Bentonite Concentration [% of the Grout Mixture Weight]	Weight Loss (WL) [%]
B1	130.0	350.0	824.9	10.0	1.4
B5	200.0		793.5	14.9	0.2

* Composition is specified per m^3^.

## Data Availability

Data are contained within the article.
